# Exploring the reasons behind nurses' intentions to leave their hospital or profession: A cross-sectional survey

**DOI:** 10.1016/j.ijnsa.2024.100232

**Published:** 2024-08-10

**Authors:** Marco Enea, Laura Maniscalco, Neeltje de Vries, Anke Boone, Olivia Lavreysen, Kamil Baranski, Silvana Miceli, Alessandra Savatteri, Walter Mazzucco, Santo Fruscione, Malgorzata Kowalska, Peter de Winter, Szymon Szemik, Lode Godderis, Domenica Matranga

**Affiliations:** a Department of Health Promotion, Mother and Child Care, Internal Medicine and Medical Specialties, University of Palermo, Palermo, Italy; b Department of Internal Medicine, Spaarne Gasthuis, Hoofddorp, the Netherlands; c Spaarne Gasthuis Academy, Hoofddorp, the Netherlands; d Centre for Environment and Health, Department of Public Health and Primary Care, KU Leuven (University of Leuven), Leuven, Belgium; e Department of Epidemiology, Medical University of Silesia, Katowice, Poland; f Department of Psychology, Educational Science and Human Movement, University of Palermo, Palermo, Italy; g Leuven Child and Health Institute, KU Leuven, Leuven, Belgium; h Department of Development and Regeneration, KU Leuven, Leuven, Belgium; i Department of Pediatrics, Spaarne Gasthuis, Haarlem and Hoofddorp, the Netherlands; j IDEWE, External Service for Prevention and Protection at Work, Interleuvenlaan 58, 3001, Heverlee, Belgium

**Keywords:** Nurses, Exhaustion, Depersonalization, Work environment, Personnel turnover, Demographic factors, Job satisfaction

## Abstract

**Background:**

Multiple factors can fuel nurses’ intention to leave their employing hospital or their profession. Job dissatisfaction and burnout are contributors to this decision. Sociodemographic and work context factors can also play a role in explaining nurses’ intention to leave.

**Objective:**

To investigate the role of sociodemographic and work context factors, including job resources, job demands, job dissatisfaction, depersonalization, and emotional exhaustion, on nurses’ intention to leave their hospital or their profession.

**Design:**

Multicentre cross-sectional study.

**Setting(s):**

Eight European hospitals, two per each country, including Belgium, the Netherlands, Italy, and Poland.

**Participants:**

From May 16 to September 30, 2022, we collected 1,350 complete responses from nurses working at the selected hospitals (13 % response rate).

**Methods:**

The intention to leave was assessed through two 5-Likert scale outcomes, agreeing with the intention to leave the profession and the intention to leave the hospital. Logistic regression models were used for statistical analysis.

**Results:**

At the multivariable analysis, a higher intention to leave the hospital was observed for: younger age, having served on the frontline against COVID-19, lack of quipment, living in the Netherlands, emotional exhaustion, dissatisfaction with work prospects, and dissatisfaction with the use of professional abilities. There was a higher intention to leave the profession for: younger age, living in the Netherlands, having work-related health problems, depersonalization, emotional exhaustion, low possibilities of professional development, dissatisfaction with work prospects, lack of use of professional abilities, overall ob issatisfaction, and dissatisfaction with salary. Nurses living in Italy expressed the lowest intention to leave.

**Conclusion:**

While confirming the role of job dissatisfaction and burnout, we found higher intention to leave for young nurses, nurses with work-related health problems, and caregivers during the COVID-19 pandemic. Dissatisfaction with work prospects, professional development, and salary also increased the intention to leave. We call for educators, managers, and policymakers to address these factors to retain at-risk nursing categories, implementing strategies to mitigate intentions to leave.

**What is already known about the topic**

• Job dissatisfaction and burnout are well-known significant predictors of the intention to leave for nurses.
• Many researchers have highlighted sociodemographic and work context factors as predictors of nurses’ intention to leave, but there are very few European studies.

Alt-text: Unlabelled box

**What this paper adds**

• This cross-sectional study on nurses’ intention to leave encompasses a range of healthcare systems within Europe.
• Young age, work-related health issues, involvement in the recent pandemic, discontent regarding career prospects, and opportunities for growth and compensation were found to be significant predictors of nurses' likelihood of leaving.

Alt-text: Unlabelled box

## 1 Introduction

In 2019–20, the nursing workforce was estimated to be 27.9 million worldwide, with an estimated global shortage of 5.9 million nurses prior to the Coronavirus Disease 2019 (COVID-19) pandemic (Buchan et al., 2022). Growing concerns about shortages in the nursing workforce have prompted proactive measures in many countries, particularly focused on increasing the training of new nurses and recruiting nurses from abroad to fill the gaps immediately (OECD, 2019; 2023). For countries belonging to the Organization for Economic Cooperation and Development, the average number of nurses per 1000 population was 9.3 in 2021 (OECD, 2023), with an increase of about 12 % over the past decade, while the mean percentage of foreign-trained nurses surpassed 9 % in 2021, with an increase of >5 % since a decade earlier. In 2021, a rapid rise in the percentage of foreign-trained nurses out of the total was recorded, for instance, with respect to the year 2012, in Canada (+27 %), United Kingdom (+53 %), Germany (+57 %), and Switzerland (+37 %) (OECD, 2023). Despite this increase, the ageing population and the rising prevalence of chronic diseases have accelerated the demand for innovative primary care models (Cristea et al., 2020; Souza et al., 2021; OECD, 2023; Choi et al., 2024). This shortage is particularly alarming as it adversely affects the accessibility and quality of healthcare services for patients (de Vries et al., 2023). Concurrently, it places an additional burden on healthcare professionals, leading to increased workload, job dissatisfaction, burnout, and a higher likelihood of professionals leaving the healthcare sector (Galanis et al., 2023), which in turn, could led to strong negative outcome for patients, such an increased inpatient hospital mortality (Catania et al., 2024). Another critical concern is the rapid ageing of the health workforce itself, aggravated by financial cutbacks, which intensifies existing migration patterns and results in severe shortages of healthcare professionals in numerous countries. Given the context of budgetary constraints and government restrictions on recruitment, there is a growing consensus on the imperative to formulate and implement effective recruitment and retention strategies for healthcare workers (WHO, 2023a).

Therefore, it is crucial to prioritize the implementation of retention policies for healthcare professionals and the identification of effective practices aimed at enhancing the resilience of healthcare workers, in accordance with the recent “Bucharest Declaration on the health and care workforce” (WHO, 2023b). These measures should enable governments to effectively address both the current and future needs of the population. To delve into the causes of the problem, several researchers investigated the determinants of nurses’ intention to leave their hospital or their profession, highlighting the role of mental health, the impact of excessive workload, and the influence of the COVID-19 outbreak (Lucchini et al., 2020; Häussl et al., 2021; Ulupınar and Erden, 2022). More recently, job satisfaction, career development and work-life balance (de Vries et al., 2023), age, number of years in the workforce, suboptimal teamwork relations (de Vries et al., 2024), and burnout (de Cordova et al., 2022; LeClaire et al., 2022) were also reported as significant factors explaining why nurses wish to stay or leave their positions.

The recent findings from a multicenter cross-sectional survey on hospital nurses from four European countries (Maniscalco et al., 2024), conducted within the framework of the project “Mental Health: Focus on Retention of Healthcare Workers”, funded by the European Health and Digital Executive Agency in 2020 as part of the 3rd European Union (EU) Health Program, contributed to shedding light on the role of mental health and wellbeing on nurses’ intention to leave. In greater detail, job resources and job demands were related to the intention to leave through the mediation of job satisfaction, work engagement, and burnout, within the framework of a job demands-job resources model. However, sociodemographic and work context characteristics were not considered in the analysis, despite their potential role for the explanation of the intention to leave. The impact of sociodemographic factors, such as age, sex, level of education, and marital status, on the stress levels of healthcare workers was also recently investigated (Nugroho et al., 2024). Of interest, age and gender, were considered variables capable of moderating the relationship between resilience and mental health indicators (Gonçalves, et al., 2022). Likewise, a supportive organization of work and work environment play a crucial role in managing stress and shaping the conflict management style among nurses (Farghaly Abdelaliem et al., 2024). A favorable work environment allows a higher quality of nursing care and job satisfaction and a lower level of burnout and staff turnover (Malinowska-Lipień et al., 2021), as well as a high-quality work organization has a negative impact on distress and sickness absenteeism (Magnavita et al., 2022).

To summarize the above findings from the literature, the hypothesis underlying this study is that some sociodemographic and work context variables prevent the intention to leave the hospital or the professional practice; others expose nurses to a higher risk. The aim of this study was to assess the impact of sociodemographic and work context variables in the explanation of the nurses’ intention to leave the hospital or professional practice. We also explored the role of job resources, job demands, job dissatisfaction, and burnout risk.

## 2 Material and methods

### 2.1 Participants and procedures

A multicenter cross-sectional study was conducted to examine the intention to leave the hospital or the profession among nurses in relation to job satisfaction, burnout, and other individual-level and hospital-level factors. The participating European hospitals were eight, two per each country (of which one was a teaching hospital), including Belgium, the Netherlands, Italy, and Poland. Data on 1350 nurses in force at each participating hospital as of April 1, 2022 were gathered between May 16 and September 30, 2022. The inclusion criteria for participants were: (1) currently working as a nurse in one of the eight participating hospitals, (2) holding a nursing degree (bachelor's or master's degree), and (3) declaring to have received adequate information about the study and willing to sign the informed consent. Potential participants were contacted by hospitals’ internal communications.

### 2.2 The questionnaire

Data were collected through an online questionnaire compliant with the General Data Protection Regulation (SurveyMonkey), which included 76 items articulated into seven sections: (1) individual information, (2) job demands; (3) job resources; (4) work engagement; (5) job satisfaction, (6) burnout, and (7) intention to leave (Maniscalco et al., 2024).

The individual information section was structured to collect information on:

• personal characteristics: nationality, sex, age, living status, education;
• work context: hospital, form of employment (permanent/temporary), type of contract (full-time/part-time) number of years of service in the hospital, nightshifts, and hospital area (medicine, surgery, emergency, and other);
• COVID-19 experience: a question to assess whether the worker was involved as a frontline with infected patients during the pandemics (Lai et al., 2020);
• working conditions: a question to assess satisfaction with the equipment available for working;
• health problems: a specific question was adapted from Eurostat's Labor Force Survey to assess the occurrence of work-related health problems in the last 3 years (EUROSTAT, 2020).

Items included in the job demands, job resources, work engagement, and job satisfaction sections were all taken from the Copenhagen Psychosocial Questionnaire (Burr et al., 2019), except staffing, which was extrapolated from the Hospital Survey on Patient Safety Culture questionnaire (Sorra et al. 2018).

The job demands section included eight domains assessed by a number of items ranging from one to four: staffing (*n* = 4), the pace of work (*n* = 4), quantitative demands (*n* = 4), cognitive demands (*n* = 4), emotional demands (*n* = 3), bullying (*n* = 1), role conflicts (*n* = 2), and work-life conflicts (*n* = 2).

The job resources section included six domains with the related items: influence at work (*n* = 4), possibility for development (*n* = 2), predictability (*n* = 2), social support from the supervisor (*n* = 2), social support from colleagues (*n* = 3), and meaning of work (*n* = 2).

The work engagement section (*n* = 3), and the job Satisfaction section (*n* = 5), included one domain each.

The burnout section was extrapolated from the Maslach Burnout Inventory (Schaufeli et al., 2001), and it included two domains: emotional exhaustion (*n* = 9) and depersonalization (*n* = 5*)*, as they are known to be associated with job demands (Zabin et al., 2023).

Last, the intention to leave (*n* = 2) section included one domain with two questions, which were the outcomes of the study: “I intend to leave my current hospital for another one in the near future” and “I intend to leave my healthcare profession for another job”, both measured in a 5-point Likert scale, from “strongly agree” to “strongly disagree” (Kaihlanen et al., 2020).

The study protocol was approved by the KU Leuven Ethical Committee (Ref. nr B3222021000679, 21 Jan 2022).

### 2.3 Measurements

The quality of the survey was measured in terms of response rate and completion rate. Methods for calculating these rates are provided in the Supplemental Material. Data records of respondents who did not accept the informed consent or abandoned the questionnaire at some point were removed from the analysis. Finally, Fisher's exact test was applied to check the assumption that the remaining missing data were missing at random. These data were imputed using the R package “mice” (van Buuren, and Groothuis-Oudshoorn, 2011), which performs multiple imputations via chained equations. Further details on data cleaning and missing data imputation are reported in the Supplemental Material.

Among the work context variables, “number of years of service in the hospital” (seniority) was categorized at 1 year of service, in part because of the collinearity with age and in part because the interest here is towards young nurses. Moreover, the variable “specialty area” included four categories: emergency, medicine, surgery, and other (Maniscalco et al., 2024).

The items for emotional exhaustion and depersonalization were scored on a 7-point Likert scale ranging between 0 and 6, as suggested by Schaufeli et al. (2001). The other items of the questionnaire, except those regarding individual information, were initially measured on a 5-point Likert scale, in agreement with the source tools (Sorra et al., 2018; Burr et al., 2019).

All 5-point items were transformed into binary variables by assigning 1 to the two highest categories (lowest in the case of reverse items) and 0 to the remaining categories in such a way as to have a positive association with the intention to leave. For example, items measured on the agreement scale (“Strongly agree”, “Agree”, “Neither agree nor disagree”, “Disagree”, and “Strongly disagree”) were dichotomized by assigning 1 to the categories “Agree” or “Strongly agree” and 0 to the others. In the case of reverse items, the value 1 was assigned to “Disagree”, or “Strongly disagree”, and 0 otherwise. A similar dichotomization was applied to items expressed on a frequency of occurrence scale (0 = ”Never” or “Seldom” or “Sometimes”; 1 = ”Often” or “Always”), extent scale (0 = “To a very small extent” or “To a small extent” or “Somewhat”; 1 = “To a large extent” or “To a very large extent”), or dissatisfaction scale (0 = “Very satisfied” or “Satisfied” or “Neither/not”; 1 = ”Very unsatisfied” or “Unsatisfied”).

All variables included in the job resources, work engagement, and job satisfaction sections were reversed in order to measure the lack of job resources, the lack of work engagement, and job dissatisfaction, respectively. For example, the original item “overall job satisfaction” included in the job satisfaction section was replaced with “overall job dissatisfaction”; the item “social support from colleagues”, included in job resources, was transformed into “lack of social support from colleagues”, and so on.

To reduce the number of binary items, the domains of the sections job demands, lack of job resources, and work engagement were summarized through composite binary indicators. and a mean score was calculated for each of these domains by averaging the binary items of each respondent. Then, the composite binary indicator of the domain was created by assigning “Yes” when the mean was above 0.5 and “No” otherwise. As an example, the domain “cognitive demands”, representing the perceived frequency of the “cognitive effort", was initially measured by four different items in a 5-point scale, and the new dichotomous variable “High Cognitive demands” was created by assigning “Yes” if the respondent's mean of the four variables was greater than 0.5, and “No” otherwise. No composite binary indicators were created for job dissatisfaction, as it was deemed preferable to keep the set of all the binary variables rather than obtaining a composite indicator for a more comprehensive analysis. Regarding the domains depersonalization and emotional exhaustion, the corresponding items were first summed (five depersonalization items scoring 0–6 were summed to obtain a composite indicator scoring in the 0–30 range, while nine emotional exhaustion items scoring 0–6 were summed to obtain a composite indicator scoring in the 0–54 range). These two composite indicators were then dichotomized, using a cut-off of 13 for depersonalization (“Yes”: score≥13, “No”: otherwise), and a cut-off of 27 for emotional exhaustion (“Yes”: score≥27, “No”: otherwise) (Dall'Ora et al., 2015).

Finally, the two outcomes measuring the intention to leave the hospital and the intention to leave the profession were dichotomized and analyzed separately by assigning the value “Yes” to the response levels “Agree” or “Strongly agree” and “No” otherwise.

### 2.4 Statistical analysis

The characteristics of the sample were reported as counts and percentages, both overall and by country, and missing data were imputed. Only age was summarized as mean and standard deviation.

The univariable analyses were carried out by measuring the association, in terms of odds ratio (OR) and *p-*value (*p*) of each independent variable with the two outcomes, through separate logistic models.

The multivariable analysis was performed through the fit of a logistic regression model for each outcome. The association between variables and each outcome was measured in terms of adjusted odds ratio (aOR) with the corresponding 95 % confidence interval (CI) and *p-*value. Because of the large number of variables, the multivariable model included only those variables resulting from a forward selection, based on the Akaike Information Criterion and performed on the set of the significant variables at the univariable analysis. A *p-*value cut-off of <0.05 was set for statistical significance. All analyses were performed using R statistical software, version 4.1.2 (R Core Team, 2023).

## 3 Results

### 3.1 Response and completion rates

Overall, the response rate was 13 % (1350 complete responses out of 10,708 potential respondents), while it was 17 % for Belgium, 9 % for the Netherlands, 8 % for Poland, and 7 % for Italy. Overall, the completion rate was 80 % (1350 out of 1680 respondents), while it was 84 % for Belgium, 80 % for the Netherlands, 65 % for Poland, and 60 % for Italy (Supplementary Material, Table A1). The flowchart of the selection of the complete responses, data cleaning, and imputation is reported in the Supplementary Material (Figure A1).

### 3.2 Descriptive analyses

The overall and by-country characteristics of survey respondents are reported in Table 1. Overall, the majority of respondent nurses were female, especially for Poland. The mean age across all participants was 44 years, with the Dutch nurses being the youngest and the Polish nurses the oldest. Approximately half of the nurses lived with their partner and children, while a little more than a quarter lived only with their partner. The vast majority of the nurses held permanent positions with almost half of them on part-time contracts. Medicine was the most common assignment hospital area. At the same time, 6 % of the nurses experienced feelings of depersonalization towards their work, and nearly one-fifth reported emotional exhaustion. Overall, almost one nurse out of ten was dissatisfied with their job.

**Table 1:** Overall and by-country numbers and percentages of survey respondents’ characteristics.

Characteristic^a^	Overall	Belgium	Italy	Netherlands	Poland
	*N*= 1350^b^	*N*= 857^b^	*N*= 86^b^	*N*= 344^b^	*N*= 63^b^
*Sociodemographic*					
Sex					
*Female*	1119 (83)	716 (84)	51 (59)	291 (85)	61 (97)
*Male*	231 (17)	141 (16)	35 (41)	53 (15)	2 (3.2)
Age (years)	43.8 (11.8)	43.7 (10.9)	45.8 (11.7)	42.6 (14.0)	50.3 (8.8)
Living status					
*Alone*	171 (13)	84 (9.8)	10 (12)	70 (20)	7 (11)
*With partner*	375 (28)	220 (26)	15 (17)	127 (37)	13 (21)
*With friends or housemates*	18 (1.3)	9 (1.1)	2 (2.3)	7 (2.0)	0 (0)
*With parents*	45 (3.3)	18 (2.1)	14 (16)	11 (3.2)	2 (3.2)
*With partner and child(ren)*	659 (49)	481 (56)	38 (44)	114 (33)	26 (41)
*Other*	82 (6.1)	45 (5.3)	7 (8.1)	15 (4.4)	15 (24)
Education					
*Bachelor's*	1147 (85)	778 (91)	76 (88)	252 (73)	41 (65)
*Master's or higher*	203 (15)	79 (9.2)	10 (12)	92 (27)	22 (35)
*Work context*					
Health problems					
*No*	848 (63)	536 (63)	58 (67)	215 (63)	39 (62)
*Yes*	502 (37)	321 (37)	28 (33)	129 (38)	24 (38)
Contract					
*Full-time*	597 (44)	363 (42)	79 (92)	92 (27)	63 (100)
*Part-time*	753 (56)	494 (58)	7 (8.1)	252 (73)	0 (0)
Employment					
*Permanent*	1296 (96)	851 (99)	61 (71)	323 (94)	61 (97)
*Temporary*	54 (4.0)	6 (0.7)	25 (29)	21 (6.1)	2 (3.2)
Seniority (years)					
≤*1 year*	77 (5.7)	35 (4.1)	7 (8.1)	34 (9.9)	1 (1.6)
*>1 year*	1273 (94)	822 (96)	79 (92)	310 (90)	62 (98)
Nightshifts					
*No*	823 (61)	631 (74)	35 (41)	133 (39)	24 (38)
*Yes*	527 (39)	226 (26)	51 (59)	211 (61)	39 (62)
COVID frontline					
*No*	323 (24)	198 (23)	29 (34)	90 (26)	6 (9.5)
*Yes*	1027 (76)	659 (77)	57 (66)	254 (74)	57 (90)
Lack of equipment					
*No*	161 (12)	84 (9.8)	28 (33)	45 (13)	4 (6.3)
*Yes*	1189 (88)	773 (90)	58 (67)	299 (87)	59 (94)
Insufficient staffing					
*No*	1162 (86)	759 (89)	68 (79)	282 (82)	53 (84)
*Yes*	188 (14)	98 (11)	18 (21)	62 (18)	10 (16)
Specialty area					
*Emergency*	142 (11)	53 (6.2)	8 (9.3)	65 (19)	16 (25)
*Medicine*	545 (40)	299 (35)	26 (30)	200 (58)	20 (32)
*Surgery*	245 (18)	152 (18)	12 (14)	77 (22)	4 (6.3)
*Other*	418 (31)	353 (41)	40 (47)	2 (0.6)	23 (37)
Academic hospital					
*No*	654 (48)	410 (48)	47 (55)	137 (40)	60 (95)
*Yes*	696 (52)	447 (52)	39 (45)	207 (60)	3 (4.8)
*Burnout*					
Depersonalization					
*No*	1268 (94)	807 (94)	82 (95)	321 (93)	58 (92)
*Yes*	82 (6.1)	50 (5.8)	4 (4.7)	23 (6.7)	5 (7.9)
Emotional exhaustion					
*No*	1111 (82)	701 (82)	58 (67)	302 (88)	50 (79)
*Yes*	239 (18)	156 (18)	28 (33)	42 (12)	13 (21)
*Job demands*					
High working pace					
*No*	624 (46)	366 (43)	32 (37)	189 (55)	37 (59)
*Yes*	726 (54)	491 (57)	54 (63)	155 (45)	26 (41)
Quantitative demands					
*No*	1096 (81)	666 (78)	74 (86)	297 (86)	59 (94)
*Yes*	254 (19)	191 (22)	12 (14)	47 (14)	4 (6.3)
Cognitive demands					
*No*	639 (47)	463 (54)	25 (29)	131 (38)	20 (32)
*Yes*	711 (53)	394 (46)	61 (71)	213 (62)	43 (68)
Emotional demands					
*No*	667 (49)	457 (53)	37 (43)	146 (42)	27 (43)
*Yes*	683 (51)	400 (47)	49 (57)	198 (58)	36 (57)
Bullying					
*No*	1121 (83)	720 (84)	62 (72)	289 (84)	50 (79)
*Yes*	229 (17)	137 (16)	24 (28)	55 (16)	13 (21)
Work-life conflicts					
*No*	1088 (81)	684 (80)	66 (77)	286 (83)	52 (83)
*Yes*	262 (19)	173 (20)	20 (23)	58 (17)	11 (17)
*Lack of work engagement*					
*No*	1297 (96)	823 (96)	82 (95)	331 (96)	61 (97)
*Yes*	53 (3.9)	34 (4.0)	4 (4.7)	13 (3.8)	2 (3.2)
*Lack of job resources*					
Lack of influence					
*No*	1081 (80)	638 (74)	74 (86)	315 (92)	54 (86)
*Yes*	269 (20)	219 (26)	12 (14)	29 (8.4)	9 (14)
Lack of development					
*No*	1298 (96)	822 (96)	75 (87)	340 (99)	61 (97)
*Yes*	52 (3.9)	35 (4.1)	11 (13)	4 (1.2)	2 (3.2)
Lack of predictability					
*No*	1182 (88)	748 (87)	54 (63)	325 (94)	55 (87)
*Yes*	168 (12)	109 (13)	32 (37)	19 (5.5)	8 (13)
Role conflicts					
*No*	1272 (94)	803 (94)	76 (88)	331 (96)	62 (98)
*Yes*	78 (5.8)	54 (6.3)	10 (12)	13 (3.8)	1 (1.6)
Lack of supervisor support					
*No*	1225 (91)	779 (91)	62 (72)	322 (94)	62 (98)
*Yes*	125 (9.3)	78 (9.1)	24 (28)	22 (6.4)	1 (1.6)
Lack of colleagues support					
*No*	1307 (97)	834 (97)	72 (84)	342 (99)	59 (94)
*Yes*	43 (3.2)	23 (2.7)	14 (16)	2 (0.6)	4 (6.3)
Lack of meaning of work					
*No*	1337 (99)	848 (99)	83 (97)	344 (100)	62 (98)
*Yes*	13 (1.0)	9 (1.1)	3 (3.5)	0 (0)	1 (1.6)
*Job dissatisfaction with:*					
Work prospects					
*No*	1187 (88)	757 (88)	73 (85)	297 (86)	60 (95)
*Yes*	163 (12)	100 (12)	13 (15)	47 (14)	3 (4.8)
Physical working conditions					
*No*	1041 (77)	667 (78)	64 (74)	252 (73)	58 (92)
*Yes*	309 (23)	190 (22)	22 (26)	92 (27)	5 (7.9)
Use of abilities					
*No*	1127 (83)	730 (85)	61 (71)	274 (80)	62 (98)
*Yes*	223 (17)	127 (15)	25 (29)	70 (20)	1 (1.6)
Overall					
*No*	1234 (91)	786 (92)	70 (81)	318 (92)	60 (95)
*Yes*	116 (8.6)	71 (8.3)	16 (19)	26 (7.6)	3 (4.8)
Salary					
*No*	756 (56)	542 (63)	19 (22)	148 (43)	47 (75)
*Yes*	594 (44)	315 (37)	67 (78)	196 (57)	16 (25)

a For each characteristic, the overall and by-country absolute frequencies are reported, as well as percentages (in brackets). The mean value is reported for Age, with the standard error (in brackets).

b N indicates the overall and by-country sample sizes.

The overall and by-country numbers and percentages of nurses’ intention to leave is reported in Table 2. The overall percentage of the intention to leave is higher regarding the profession than the hospital. In both cases, the Netherlands had the highest prevalence, while Poland had the lowest.

**Table 2:** Overall and by-country numbers and percentages of nurses’ intention to leave with 95 % CI (Confidence Interval).

		Intention to leave the hospital			Intention to leave the profession		
Country	*N*	Yes	%	95 % CI	Yes	%	95 % CI
Belgium	857	54	6.3	6.9 - 9.9	108	12.6	10.4 - 14.8
Italy	86	7	8.1	2.3 - 13.9	6	7.0	1.6 - 12.4
Netherlands	344	50	15.0	10.8 - 18.2	66	19.2	15.0 - 23.4
Poland	63	2	3.2	0 - 7.5	4	6.3	0.3 - 12.3
Overall	1350	113	8.4	6.9 - 9.9	184	13.6	11.7 - 15.4

### 3.3 Univariable analysis

The univariable analysis of each independent variable with respect to the intention to leave the hospital or the profession is reported in Table 3. Specifically for nurses who expressed a higher intention to leave the hospital, statistically significant sociodemographic variables were: males, young age, Dutch nationality (by using Belgian as reference in the logit model), and having a master's degree. The working context factors were: working in an emergency area as compared to working in another healthcare setting, nightshifts, frontline service during COVID-19 pandemic, experiencing equipment shortages, and reporting work-related health problems. With regard to burnout, nurses experiencing emotional exhaustion and depersonalization showed higher intention to leave. Among the significant job demands, there were quantitative demands, experiencing bullying, and work-life conflicts. Among the variables measuring the lack of job resources, higher intention to leave the hospital were observed for the lack of development opportunities, the lack of predictability, role conflicts, the lack of a supervisor support, and the lack of meaning of work. A lack of work engagement was positively associated with the intention to leave the hospital. Finally, among the variables measuring job dissatisfaction, those statistically significant were dissatisfaction with work prospects, with physical working conditions, with the use of professional, with salary, and overall job dissatisfaction.

**Table 3:** Univariable analysis of each independent variable with respect to the nurses’ intention to leave the hospital or the profession.

	Intention to leave the hospital				Intention to leave the profession			
Characteristics^a^	No	Yes	OR	*p*	No	Yes	OR	*p*
*Socio-demographic*								
Sex								
*Female*	1037 (92.7)	82 (7.3)	–	–	973 (87)	146 (13)	–	–
*Male*	200 (86.6)	31 (13.4)	1.96	**0.003**	193 (83.5)	38 (16.5)	1.31	0.171
Age								
Mean (SD)	44.4 (11.8)	37.6 (10.1)	0.95*	**<0.001**	44.7 (11.8)	38.2 (10.3)	0.95*	**<0.001**
Country								
*Belgium*	803 (93.7)	54 (6.3)	–	–	749 (87.4)	108 (12.6)	–	–
*Italy*	79 (91.9)	7 (8.1)	1.32	0.51	80 (93)	6 (7)	0.52	0.133
*Netherlands*	294 (85.5)	50 (14.5)	2.53	**<0.001**	278 (80.8)	66 (19.2)	1.65	**0.004**
*Poland*	61 (96.8)	2 (3.2)	0.49	0.327	59 (93.7)	4 (6.3)	0.47	0.152
Education								
*Bachelor*	1059 (92.3)	88 (7.7)	–	–	995 (86.7)	152 (13.3)	–	–
*Master or higher*	178 (87.7)	25 (12.3)	1.69	**0.029**	171 (84.2)	32 (15.8)	1.22	0.337
Living status								
*Alone*	157 (91.8)	14 (8.2)	–	–	144 (84.2)	27 (15.8)	–	–
*With partner*	342 (91.2)	33 (8.8)	1.08	0.813	328 (87.5)	47 (12.5)	0.76	0.304
*With friends or* *housemates*	15 (83.3)	3 (16.7)	2.24	0.243	16 (88.9)	2 (11.1)	0.67	0.603
*With parents*	41 (91.1)	4 (8.9)	1.09	0.880	37 (82.2)	8 (17.8)	1.15	0.748
*With partner and* *child(ren)*	605 (91.8)	54 (8.2)	1	0.998	571 (86.6)	88 (13.4)	0.82	0.412
*Other*	77 (93.9)	5 (6.1)	0.73	0.556	70 (85.4)	12 (14.6)	0.91	0.812
*Work context*								
Health problems								
*No*	790 (93.2)	58 (6.8)	–	–	768 (90.6)	80 (9.4)	–	–
*Yes*	447 (89)	55 (11)	1.68	**0.009**	398 (79.3)	104 (20.7)	2.51	**<0.001**
Nightshifts								
*No*	771 (93.7)	52 (6.3)	–	–	732 (88.9)	91 (11.1)	–	–
*Yes*	466 (88.4)	61 (11.6)	1.94	**0.001**	434 (82.4)	93 (17.6)	1.72	**0.001**
Contract								
*Full-Time*	550 (92.1)	47 (7.9)	–	–	525 (87.9)	72 (12.1)	–	–
*Part-Time*	687 (91.2)	66 (8.8)	1.12	0.557	641 (85.1)	112 (14.9)	1.27	0.135
Employment								
*Permanent*	1190 (91.8)	106 (8.2)	–	–	1122 (86.6)	174 (13.4)	–	–
*Temporary*	47 (87)	7 (13)	1.67	0.218	44 (81.5)	10 (18.5)	1.47	0.288
COVID frontline								
*No*	310 (96)	13 (4)	–	–	296 (91.6)	27 (8.4)	–	–
*Yes*	927 (90.3)	100 (9.7)	2.57	**0.002**	870 (84.7)	157 (15.3)	1.98	**0.002**
Lack of equipment								
*No*	1110 (93.4)	79 (6.6)	–	–	1044 (87.8)	145 (12.2)	–	–
*Yes*	127 (78.9)	34 (21.1)	3.76	**<0.001**	122 (75.8)	39 (24.2)	2.30	**<0.001**
Insufficient staff								
*No*	1068 (91.9)	94 (8.1)	–	–	1003 (86.3)	159 (13.7)	–	–
*Yes*	169 (89.9)	19 (10.1)	1.28	0.355	163 (86.7)	25 (13.3)	0.97	0.886
Specialty area								
*Other*	392 (93.8)	26 (6.2)	–	–	213 (86.9)	32 (13.1)	–	–
*Emergency*	120 (84.5)	22 (15.5)	2.76	**0.001**	110 (77.5)	32 (22.5)	2.03	**0.003**
*Medicine*	493 (90.5)	52 (9.5)	1.59	0.063	476 (87.3)	69 (12.7)	1.04	0.831
*Surgery*	232 (94.7)	13 (5.3)	0.84	0.630	367 (87.8)	51 (12.2)	1.08	0.747
University hospital								
*No*	604 (92.4)	50 (7.6)	–	–	569 (87)	85 (13)	–	–
*Yes*	633 (90.9)	63 (9.1)	1.2	0.352	597 (85.8)	99 (14.2)	1.11	0.511
*Burnout*								
Depersonalization								
*No*	1177 (92.8)	91 (7.2)	–	–	1129 (89)	139 (11)	–	–
*Yes*	60 (73.2)	22 (26.8)	4.74	**<0.001**	37 (45.1)	45 (54.9)	9.88	**<0.001**
Emotional exhaustion								
*No*	1046 (94.1)	65 (5.9)	–	–	1020 (91.8)	91 (8.2)	–	–
*Yes*	191 (79.9)	48 (20.1)	4.04	**<0.001**	146 (61.1)	93 (38.9)	7.14	**<0.001**
*Job demands*								
High pace								
*No*	578 (92.6)	46 (7.4)	–	–	556 (89.1)	68 (10.9)	–	–
*Yes*	659 (90.8)	67 (9.2)	1.28	0.220	610 (84)	116 (16)	1.55	**0.007**
Quantitative demands								
*No*	1014 (92.5)	82 (7.5)	–	–	962 (87.8)	134 (12.2)	–	–
*Yes*	223 (87.8)	31 (12.2)	1.72	**0.015**	204 (80.3)	50 (19.7)	1.76	**0.002**
Cognitive demands								
*No*	587 (91.9)	52 (8.1)	–	–	562 (87.9)	77 (12.1)	–	–
*Yes*	650 (91.4)	61 (8.6)	1.06	0.770	604 (85)	107 (15)	1.29	0.109
Emotional demands								
*No*	616 (92.4)	51 (7.6)	–	–	597 (89.5)	70 (10.5)	–	–
*Yes*	621 (90.9)	62 (9.1)	1.21	0.343	569 (83.3)	114 (16.7)	1.71	**0.001**
Bullying								
*No*	1038 (92.6)	83 (7.4)	–	–	986 (88)	135 (12)	–	–
*Yes*	199 (86.9)	30 (13.1)	1.89	**0.005**	180 (78.6)	49 (21.4)	1.99	**<0.001**
Work-life conflicts								
*No*	1015 (93.3)	73 (6.7)	–	–	989 (90.9)	99 (9.1)	–	–
*Yes*	222 (84.7)	40 (15.3)	2.51	**<0.001**	177 (67.6)	85 (32.4)	4.8	**<0.001**
*Lack of Job Resources*								
Lack of influence								
*No*	989 (91.5)	92 (8.5)	–	–	946 (87.5)	135 (12.5)	–	–
*Yes*	248 (92.2)	21 (7.8)	0.91	0.709	220 (81.8)	49 (18.2)	1.56	**0.015**
Lack of development								
*No*	1197 (92.2)	101 (7.8)	–	–	1130 (87.1)	168 (12.9)	–	–
*Yes*	40 (76.9)	12 (23.1)	3.56	**<0.001**	36 (69.2)	16 (30.8)	2.99	**<0.001**
Lack of predictability								
*No*	1099 (93)	83 (7)	–	–	1041 (88.1)	141 (11.9)	–	–
*Yes*	138 (82.1)	30 (17.9)	2.88	**<0.001**	125 (74.4)	43 (25.6)	2.54	**<0.001**
Role conflicts								
*No*	1176 (92.5)	96 (7.5)	–	–	1118 (87.9)	154 (12.1)	–	–
*Yes*	61 (78.2)	17 (21.8)	3.41	**<0.001**	48 (61.5)	30 (38.5)	4.54	**<0.001**
Lack of supervisor support								
*No*	1140 (93.1)	85 (6.9)	–	–	1078 (88)	147 (12)	–	–
*Yes*	97 (77.6)	28 (22.4)	3.87	**<0.001**	88 (70.4)	37 (29.6)	3.08	**<0.001**
Lack of colleagues support								
No	1199 (91.7)	108 (8.3)	–	–	1130 (86.5)	177 (13.5)	–	–
Yes	38 (88.4)	5 (11.6)	1.46	0.436	36 (83.7)	7 (16.3)	1.24	0.607
Lack of meaning of work								
*No*	1228 (91.8)	109 (8.2)	–	–	1158 (86.6)	179 (13.4)	–	–
*Yes*	9 (69.2)	4 (30.8)	5.01	**0.008**	8 (61.5)	5 (38.5)	4.04	**0.015**
*Lack of work engagement*								
*No*	1199 (92.4)	98 (7.6)	–	–	1140 (87.9)	157 (12.1)	–	–
*Yes*	38 (71.7)	15 (28.3)	4.83	**<0.001**	26 (49.1)	27 (50.9)	7.54	**<0.001**
*Job dissatisfaction with:*								
Work prospects								
*No*	1122 (94.5)	65 (5.5)	–	–	1071 (90.2)	116 (9.8)	–	–
*Yes*	115 (70.6)	48 (29.4)	7.2	**<0.001**	95 (58.3)	68 (41.7)	6.61	**<0.001**
Physical working conditions								
*No*	982 (94.3)	59 (5.7)	–	–	948 (91.1)	93 (8.9)	–	–
*Yes*	255 (82.5)	54 (17.5)	3.52	**<0.001**	218 (70.6)	91 (29.4)	4.26	**<0.001**
Use of abilities								
*No*	1069 (94.9)	58 (5.1)	–	–	1021 (90.6)	106 (9.4)	–	–
*Yes*	168 (75.3)	55 (24.7)	6.03	**<0.001**	145 (65)	78 (35)	5.18	**<0.001**
Overall								
*No*	1155 (93.6)	79 (6.4)	–	–	1108 (89.8)	126 (10.2)	–	–
*Yes*	82 (70.7)	34 (29.3)	6.06	**<0.001**	58 (50)	58 (50)	8.79	**<0.001**
Salary								
*No*	711 (94)	45 (6)	–	–	696 (59.7)	60 (32.6)	–	–
*Yes*	526 (88.6)	68 (11.4)	2.04	**<0.001**	470 (40.3)	124 (67.4)	3.06	**<0.001**

a For each category of the characteristics, the intention to leave is showed in terms of absolute frequencies and percentages (in brackets) and the first category is the reference one. Mean and standard deviation (SD) are reported for Age. OR: odds ratio Statistically significant p-values (*p*) are highlighted in bold-type. *The OR for Age is meant with respect one-year increases.

Specifically for nurses who expressed a higher intention to leave the profession, statistically significant sociodemographic variables were young age and Dutch nationality. The working context factors were: reporting work-related health problems, working in an emergency area, nightshifts, frontline service during COVID-19 pandemic, and experiencing equipment shortages. With regard to burnout, nurses experiencing emotional exhaustion and depersonalization showed higher intention to leave. Among the significant job demands, there were: working at a high pace, quantitative demands, emotional demands, experiencing bullying, and work-life conflicts. Among the variables measuring the lack of job resources, higher intention to leave the profession were observed for the lack of influence, the lack of development opportunities, the lack of predictability, role conflicts, the lack of a supervisor support, and the lack of meaning of work. A lack of work engagement was positively associated with the intention to leave the profession. Finally, among the variables measuring job dissatisfaction, those statistically significant were: dissatisfaction with work, with physical working conditions, with the use of professional abilities, with salary, and the overall job dissatisfaction.

### 3.4 Multivariable analysis

The estimates of the multivariable logistic models for the intention to leave the hospital or the healthcare profession are shown in Table 4. The models highlighted several significant determinants commonly associated with the intention to leave both the hospital and the profession: young age, living in the Netherlands, experiencing emotional exhaustion, and dissatisfaction with work prospects and with use of professional abilities.

**Table 4:** Multivariable logistic model estimates for the nurses’ intention to leave the hospital or the healthcare profession.

	**Intention to leave the hospital**				**Intention to leave the profession**			
	95 % CI				95 % CI			
**Characteristics**^a^	aOR	2.5 %	97.5 %	*p*	aOR	2.5 %	97.5 %	*p*
*Sociodemographic*								
Sex Male	1.59	0.92	2.67	0.087	–	–	–	–
Age	0.95	0.94	0.97	**<0.001**	0.95	0.94	0.97	**<0.001**
Country (vs Belgium)								
Italy	0.46	0.15	1.21	0.137	0.17	0.05	0.44	**0.001**
Netherlands	3.15	1.96	5.07	**<0.001**	1.78	1.18	2.70	**0.006**
Poland	0.67	0.09	2.94	0.645	0.60	0.14	1.92	0.446
*Work context*								
COVID frontline	2.06	1.11	4.13	**0.030**	1.44	0.87	2.45	0.163
Lack of equipment	2.18	1.26	3.70	**0.005**	–	–	–	–
Health problems	–	–	–	–	1.49	1.01	2.18	**0.044**
*Burnout*								
Depersonalization	–	–	–	–	2.58	1.43	4.66	**0.002**
Emotional exhaustion	2.59	1.57	4.22	**<0.001**	3.05	1.88	4.94	**<0.001**
*Lack ofjob resources*								
Lack of development	2.2	0.89	5.12	0.076	2.71	1.16	6.05	**0.018**
Lack of supervisor support	1.8	0.98	3.23	0.051	–	–	–	–
Work-life conflicts	–	–	–	–	1.51	0.96	2.35	0.071
*Job dissatisfaction with:*								
Work prospects	2.68	1.58	4.52	**<0.001**	1.84	1.10	3.04	**0.018**
Use of abilities	2.56	1.54	4.2	**<0.001**	2.1	1.33	3.27	**<0.001**
Overall	–	–	–	–	2.11	1.2	3.69	**0.009**
Salary	–	–	–	–	1.91	1.29	2.84	**0.001**

a For each characteristic and model, adjusted odds ratio (aOR), confidence interval (CI), and *p*-value (*p*) are reported. Significant *p*-values are highlighted in bold type.

Specifically for the intention to leave the hospital, there was a significantly higher risk for nurses who served on the frontline during the COVID-19 and experienced lack of equipment.

Specifically for the intention to leave the profession, there was a significantly lower risk for Italian nurses compared to Belgian ones, and a significantly higher risk for nurses reporting health problems, depersonalization, lack of development opportunities, job dissatisfaction, and dissatisfaction with salary.

## 4 Discussion

This paper is the second in a sequence of publications presenting the findings of our survey. In the initial paper, we discussed the direct and indirect factors influencing intentions to leave among both physicians and nurses, utilizing the job demands-resources model (Maniscalco et al., 2024). In this current study, we narrowed its focus to nurses, shedding light on the impact of both individual and hospital-level factors. We not only reaffirmed the significance of burnout and job satisfaction but also uncovered the importance of factors such as age, experience with COVID-19 patients, previous health issues, and nationality as critical individual determinants, alongside the notable influence of equipment shortages as a key hospital-related factor.

The intention to leave the nursing profession, more than the intention to leave the hospital, could be attributed to the unbalance between exerted work efforts and received rewards. Working and organizational conditions, particularly exacerbated during the pandemic, were profoundly dissatisfying and inadequately compensated. These circumstances failed to incentivize nurses to remain within the profession, prompting them to consider alternative career paths or life choices.

Burnout was found to be the primary determinant correlated with the nurse's intention to leave the profession (through the two components emotional exhaustion and depersonalization) or the hospital (emotional exhaustion only). In Ulupınar and Erden (2022), depersonalization was found to be a direct determinant of the nurses’ intention to leave, while emotional exhaustion acted only indirectly through its correlation with job demands and depersonalization. As discussed elsewhere (Gago-Valiente et al., 2021), feeling exhausted could be experienced as an intrinsic and unavoidable feature of nursing activity in the hospital setting, while depersonalization can be an alarm bell for major depression.

Among the sociodemographic factors, age played a significant role in both outcomes, as the younger nurses were more likely to consider leaving both the hospital and the healthcare profession. This result is in line with a recent systematic review suggesting that young nurses’ limited work experience could be responsible for a minor resilience to stress and high work pace (de Vries et al., 2023). However, this association is primarily debated in the literature, as many authors claim that both young and old healthcare workers intend to leave to the same extent (Koch et al., 2020; Peter et al., 2020).

Dutch nurses were found to be significantly more intent on leaving both the hospital and the profession than the Belgian nurses, while Italian nurses showed the smallest intention to leave the profession. These differences might be attributed to multiple factors, such as salary levels adjusted for cost of living, labor market conditions, and higher education levels. Being unsatisfied with salary increased the intention to leave the hospital or the profession. Earlier researchers have shown a correlation between salary and intention to leave (Domagała and Dubas-Jakóbczyk, 2019; Karlsson et al., 2019), and Dutch nurses have lower salaries (adjusted for cost of living) than Belgian colleagues (OECD, 2022).

Another hypothesis on why Dutch nurses had higher intention to leave regards the most favorable labor market conditions, in terms of the higher employment rate in the health and social work sectors (OECD, 2023), and of the higher overall employment rate (EUROSTAT, 2024), which can paradoxically lead to higher turnover intentions, as nurses know the abundance of alternative job opportunities within and outside the healthcare sector. In Italy, the even-more-rigid healthcare labor market could explain why the observed tendency of Italian nurses to leave the profession is lower. However, nowadays in Italy, we are witnessing the beginning of a process of transformation in favour of greater flexibility, with an increase in transfers, job relocations, public-private transfers, and the continuous search for better working conditions, not only as salary growth but also opportunities for general conditions and quality of service, prospects for growth, new training needs, and modern technological supports (Cirillo et al., 2017). Higher-educated nurses could have a higher intention to leave (de Vries, 2023), especially if their work expectations are not satisfied. From our sample's composition, we found higher master's degree percentages for Dutch nurses than Belgian and Italian nurses, though their education was lower than Polish nurses. Moreover, among the countries considered in this paper, the Netherlands has the highest percentage of graduated nurses (OECD, 2021). It can be hypothesized that the major tendency to leave reported by Dutch nurses could result from the underutilization of skills that higher-educated nurses undergo in a work environment where they often perform the same duties as those with lower education levels. The lack of diverse and more complex technical nursing tasks, as well as the absence of professional opportunities, such as for clinical leadership or innovation (Kox et al., 2020), could be an explanation for why this subcategory had a higher intention to leave, which is also confirmed by earlier research (de Vries et al., 2023). Additionally, the reported heterogeneity of the intention to leave across countries is not uncommon (Heinen et al., 2013) and could depend on multiple country-level and hospital-level factors, probably not all observed in this study.

The important result of higher intention to leave the hospital for nurses who served on the frontline against COVID-19 is consistent with previous studies (de Vries et al., 2024). There is evidence of the increased prevalence of intention to leave during COVID-19 compared to the pre-pandemic period. It can be hypothesized that the contagion fear, anxiety, and burnout symptoms due to the pressing work pace in the ward, as well as the isolation from family, could strengthen the intention to leave the hospital (de Vries et al., 2024).

Another notable result was the significantly elevated risk of intention to leave the profession for nurses with a history of work-related health problems. This finding aligns with a systematic review emphasizing the impact of health on the intention to leave and underscores the importance of organizational factors, such as leadership in supporting nurses (Tolksdorf et al., 2022).

Being unsatisfied because of work prospects and dissatisfaction with the use of professional abilities was found to be an important predictor of the intention to leave the hospital, while overall job dissatisfaction was a significant determinant of the intention to leave the professional practice. Of note, nurses having a lower quality of life at work had more intention to leave their organization, and the main reasons included dissatisfaction with workplace safety and cleanliness, career prospects, poor support by supervisors, and dissatisfaction with wages (Al Zamel et al., 2020). Although these specific features of job dissatisfaction could induce nurses to leave their hospital, it is necessary to be entirely dissatisfied for intending to leave the profession. Conversely, nurses with higher intention to leave the profession reported limited possibilities for development, including opportunities for ongoing training and promotion, as a significant factor for nurses’ job satisfaction and intention to stay (Shiri et al., 2023), while other researchers did not report such an association (Moloney et al., 2018).

Among hospital-related determinants, a lack of equipment was significantly associated with the intention to leave the profession. There is evidence that the shortage of supplies and the malfunction or lack of personal protective equipment, which were exacerbated during the COVID-19 pandemic, are related to distress (Martin et al., 2023), burnout, and intention to leave the job and the profession (Bruyneel et al., 2023; de Vries et al., 2024).

This study has several strengths and weaknesses. The first strength lies in the sample composition. Despite being not randomly selected, the chosen countries were strategically representative of various aspects of the EU, offering insight into Europe's intrinsic heterogeneity in terms of geography, healthcare systems, gross domestic product per capita, and nurse-to-population ratios. Of note, in 2020, the nurse-to-population ratio, per 1000 population, was above the EU mean (8.3) for Belgium (11.1) and the Netherlands (11.1), and below the EU mean for Poland (5.1) and Italy (6.3) (OECD/European Union, 2022).

Further, in this study, we addressed relevant aspects of job dissatisfaction associated with intention to leave, providing knowledge that can steer the policymakers to design and adopt tailored retention policies and interventions.

Among the study limitations, there are the cross-sectional design, which prevented us from inferring causal relations. Furthermore, selection bias that can occur if the most motivated nurses to join the survey showed different likelihood of intention to leave. Additionally, it was not possible to further investigate on the effect of country-level variables because of the low number of countries involved in the survey. Indeed, including country-level variables in our model would have provided collinearity, lack of degrees of freedom, and ecological bias. Moreover, although a question about satisfaction with salary was included, the lack of quantitative information about remuneration, income, and salary retention schemes would have been a valuable confounder to be considered in the analysis. Lastly, the low response rate suggests caution regarding the generalizability of the results.

## 5 Conclusion

Effectively intervening on the intention to leave the profession or the hospital context represents a real challenge for the healthcare system. This requires a profound change within the system, which leads to the development of a workplace that promotes greater job satisfaction. If it is true that the recent COVID-19 pandemic has significantly increased the dissatisfaction of nurses, at the same time this phenomenon has deep origins and requires the development of targeted and functional policies. These policies can be specific to the hospital and personnel category and may include offering competitive salaries, creating a positive work environment, allowing a good work-life balance, assigning fair workloads, and acknowledging of staff professional roles by supervisors.

We actively explored the determinants influencing nurses' intentions to leave their profession or hospital, focusing on both individual and institutional factors. Notably, we identified young nurses, those whose health has worsened due to their work, and nurses who served during recent pandemics as being at greater risk. Moreover, complete dissatisfaction with the job, along with specific discontent regarding career prospects, opportunities for growth, and compensation, significantly heightened nurses' likelihood of leaving. Educators, managers, and policymakers should pay close attention to these critical determinants to enhance job retention and develop strategies that reduce the turnover risk among these vulnerable nurse groups.

## Funding

External funding was received for the development of this study: European Union, HaDEA (Grant Agreement (GA) No: 101018310)—3rd Health Programme, Multibeneficiary Project Grant (HP-PJ, HP-JA), Topic: PJ-01- 2020-1, Type of action: HP-PJ, SEP-210693712: Project called METEOR (MEnTal hEalth: fOcus on Retention of healthcare workers). The funding source did not influence the study design, the collection, analysis and interpretation of the data, the manuscript writing, or the decision to submit the manuscript for publication. Both the views that are expressed and any errors or omissions are the sole responsibility of the authors.

## CRediT authorship contribution statement

**Marco Enea:** Writing – original draft, Methodology, Formal analysis, Data curation, Conceptualization. **Laura Maniscalco:** Writing – original draft, Methodology, Formal analysis, Data curation, Conceptualization. **Neeltje de Vries:** Writing – review & editing, Investigation. **Anke Boone:** Writing – review & editing, Investigation. **Olivia Lavreysen:** Writing – review & editing, Investigation. **Kamil Baranski:** Writing – review & editing, Investigation. **Silvana Miceli:** Writing – review & editing. **Alessandra Savatteri:** Investigation. **Walter Mazzucco:** Writing – review & editing, Investigation. **Santo Fruscione:** Investigation. **Malgorzata Kowalska:** Writing – review & editing, Investigation. **Peter de Winter:** Writing – original draft, Methodology. **Szymon Szemik:** Writing – review & editing, Methodology. **Lode Godderis:** Writing – review & editing, Methodology, Funding acquisition. **Domenica Matranga:** Writing – original draft, Supervision, Methodology, Formal analysis, Data curation, Conceptualization.

## Declaration of competing interest

The authors declare that they have no known competing financial interests or personal relationships that could have appeared to influence the work reported in this paper.

## Data Availability

The datasets used and analyzed during the current study are available from the corresponding author on reasonable request.

## Appendix Supplementary materials

Image, application 1
