# Supplementary material for: Exploring the reasons behind nurses' intentions to leave their hospital or profession: A cross-sectional survey
Source: Int J Nurs Stud Adv. 2024 Aug 10;7:100232. doi: 10.1016/j.ijnsa.2024.100232 (PMC11367642; doi:10.1016/j.ijnsa.2024.100232)
Supplement: Supplementary file 1 [file mmc1.docx]

**Supplementary Material**

to the paper:

**Exploring the Reasons Behind Nurses' Intentions to Leave Their Hospital or Profession: A Cross-Sectional Survey**

Marco Enea^§1*^, Laura Maniscalco^§1^, Neeltje de Vries^2,3^, Anke Boone^4^, Olivia Lavreysen^4^, Kamil Baranski^5^, Silvana Miceli^6^, Alessandra Savatteri^1^, Walter Mazzucco^1^, Santo Fruscione^1^, Malgorzata Kowalska^5^, Peter de Winter^7,8,9^, Szymon Szemik^5^, Lode Godderis^4,10^, and Domenica Matranga ^1^

**^§^**these authors contributed equally. *Correspondence to: [marco.enea@unipa.it](mailto:marco.enea@unipa.it)

**Affiliations**

1 – Department of Health Promotion, Maternal and Infant Care, Internal Medicine and Medical Specialties, University of Palermo, Palermo, Italy

2 – Department of Internal Medicine, Spaarne Gasthuis, Hoofddorp, The Netherlands

3 – Spaarne Gasthuis Academy, Hoofddorp, The Netherlands

4 – Centre for Environment and Health, Katholieke University, Leuven, Belgium

5 – Department of Epidemiology, Medical University of Silesia, Katowice, Poland

6 – Department of Psychology, Educational Science and Human Movement, University of Palermo, Palermo, Italy

7 – Leuven Child and Health Institute, KU Leuven, Leuven, Belgium

8 – Department of Development and Regeneration, KU Leuven, Leuven, Belgium

9 – Department of Pediatrics, Spaarne Gasthuis, Haarlem and Hoofddorp, The Netherlands

10 - IDEWE, External Service for Prevention and Protection at Work, Interleuvenlaan 58, 3001, Heverlee, Belgium

***1. Data quality assessment***

The quality of the METEOR survey for nurses is measured by reporting the Response rate and the Completion rate, measured as:

$Response rate=\frac{number of completed surveys}{number of email sent}$

$Completion rate=\frac{number of completed surveys}{number of respondents who entered the survey}$

The “number of completed surveys” is what we get once that incomplete, inappropriate, and anomalous response patterns records are removed. The “number of emails sent” corresponds, for the METEOR survey, to the total number of physicians and nurses working in all the eight participating hospitals. The “number of respondents who entered the survey” are calculated as the total number of clicks on the invitation link.

**Table A1. Overall and by country Response and Completion rates for the METEOR survey for physicians and nurses.**

| Country | N. of nurses  (a) | N. of respondents  (b) | N. of complete responses  (c) | Response rate  (d = c / a) | Completion rate  (e = c / b) |
| --- | --- | --- | --- | --- | --- |
| Belgium | 4980 | 1014 | 852 | 17% | 84% |
| Netherlands | 3707 | 429 | 345 | 9% | 80% |
| Italy | 1212 | 141 | 85 | 7% | 60% |
| Poland | 809 | 96 | 62 | 8% | 65% |
| **Overall** | **10708** | **1680** | **1350** | **13%** | **80%** |

*Data quality check*

The overall number of nurses who answered the questionnaire was 1350, out of 10708 potential respondents, with a 13% Response rate, while 1350 out of 1680 completed the questionnaire, with an 80% Completion rate. Belgium provided the highest Response rate (17%) and the highest Completion rate (84%), while Italy has the lowest Response (7%) and Completion rates (60%) (Table A1).

Of 1680 participants, 330 were discharged for several reasons: respondents either did not accept the informed consent (57) or, if they did, they immediately abandoned the questionnaire without filling in any response (31) or abandoned the questionnaire at some point (251). We also removed one data record as the identity of that respondent was at risk. The percentage of missingness per variable was very low: 3.9% for the variable specialty area, 2.1% per education, 0.7% per sex, and a range of 0%-0.5% for all other variables. Eventually, the total number of records used to estimate the models was 1350. These were assessed for data quality by checking at the possible presence of unlikely response patterns, but no occurrence of this type was revealed. The checked dataset was then used to perform a descriptive analysis of the sample collected. A total of 1111 records did not contain any missing data in the variables of interest, while the remaining 239 records that showed missing data were assumed to be “missing completely at random” (MCAR). Such an assumption was verified by performing the Fisher’s exact test: for each of the response variables, and for each independent variable presenting at least 5 missing data, possible significant differences between the complete data group and the missing data one, were assessed. Finally, the incomplete data records were imputed using the R package “mice” (van Buuren and S. Groothuis-Oudshoorn, 2011), which performs multiple imputations via chained equations (Figure A1).


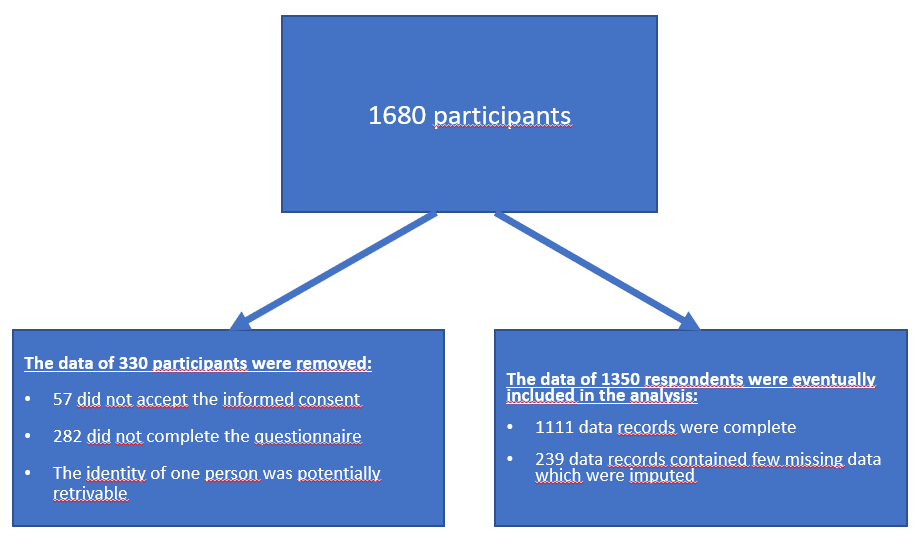


Figure A1. Flowchart of nurses' data cleaning and imputation.

**References**

van Buuren, S. Groothuis-Oudshoorn, SK (2011). mice: Multivariate Imputation by Chained Equations in R. *J Stat Softw*; 45(3), 1-67
